# Phytochemical Analysis and Antioxidant Activity of Centella Asiatica Extracts: An Experimental and Theoretical Investigation of Flavonoids

**DOI:** 10.3390/plants12203547

**Published:** 2023-10-12

**Authors:** Anbazhakan Kandasamy, Kanakaraj Aruchamy, Praveena Rangasamy, Deepha Varadhaiyan, Chandrasekar Gowri, Tae Hwan Oh, Subramaniyan Ramasundaram, Balasankar Athinarayanan

**Affiliations:** 1 Department of Physics, Gobi Arts & Science College, Gobichettipalayam, Erode 638453, India; aganphysics@gmail.com; 2 School of Chemical Engineering, Yeungnam University, Gyeongsan 38541, Republic of Korea; a.kanakaraj@yu.ac.kr (K.A.); taehwanoh@ynu.ac.kr (T.H.O.); 3 Department of Chemistry, Bannari Amman Institute of Technology, Sathyamangalam 638401, India; deephav@bitsathy.ac.in; 4 Sri Shanmugha College of Engineering and Technology, Pullipalayam, Salem 637304, India; chandrudgs@gmail.com

**Keywords:** *C. asiatica*, GC-MS, antioxidant activity, density functional theory, molecular docking

## Abstract

Centella asiatica (CA) is a medicinal plant widely used in the East, with many of its phytoconstituents remaining unexplored. In this study, compounds were extracted and identified from *C. asiatica* to determine its medicinal properties. Phytochemical screening was conducted on shoot, callus, and cell suspension extracts, revealing the presence of tannins, flavonoids, terpenoids, saponins, and steroids in all three cultures, with no alkaloids detected. IC50 values were determined to evaluate the antioxidant activity of the extracts, with the highest value observed for cell suspension culture (20 µg/mL), followed by shoot culture (19 µg/mL), and then callus extract (10 µg/mL), with ascorbic acid as the standard at an IC50 value of 26.25 µg/mL. Finally, density functional theory was used to analyze the structure–activity relationships of the identified compounds from *C. asiatica* extract. The results suggest that ultrasonic-assisted extraction yielded the highest recovery and antioxidant activity, with a scavenging activity of 79%. This study provides valuable insights into the phytochemical composition and antioxidant potential of *C. asiatica*, which may have implications for its use in traditional medicine and future drug development.

## 1. Introduction

Centella asiatica (*C. asiatica*), vernacularly referred to as gotu kola or vallarai, is a tropical herbaceous plant that is a member of the *Umbelliferae (Apiaceae*) family and is indigenous to Asian nations such as India, Japan, China, Indonesia, Sri Lanka, and South Africa [1]. It belongs to the parsley family and has tiny green fan-shaped leaves, white or light purple to pink blossoms, and little oval fruits. It has no flavor or aroma. The Indian Pharmacopeia states that *C. asiatica* has a wide range of medicinal and therapeutic benefits [2] on conditions like leprosy, lupus, varicose ulcers, eczema, psoriasis, diarrhea, fever, and amenorrhea [3]. The pharmacological effects of *C. asiatica*, including its antioxidant, antibacterial, anti-inflammatory [4], neuroprotective, memory-improving [5], and depressive substances [6], have also been covered in recent studies. Due to its well-documented therapeutic advantages [7], it has increasingly been included in the pharmacopoeias of various countries, including China, England, France, Italy, the Netherlands, Germany, and Poland [8]. Secondary metabolites, which make up the majority of *C. asiatica*’s biologically effective components, were mostly extracted from the aerial sections. There are 57 secondary metabolites in total that have been identified in *C. asiatica*, and they can be divided into four main groups based on their chemical composition: triterpenoids [9], volatile mono- and sesquiterpenes, flavonoids, and other compounds (vellarine and hydrocotyline). The most prevalent pentacyclic triterpenoids, asiaticoside and madecassoside, as well as their corresponding madecassic acid and asiatic acid, and aglycones have all undergone substantial research and are now recognized as the main active ingredients of *C. asiatica* [10]. Due to the high moisture content of herbaceous plants, including *C. asiatica*, post-harvest drying is necessary to regulate the moisture level, minimize nutrition loss, and promote their supply during the off-season [11].

However, due to various conditions, including thermal treatment and oxidation reactions during processing, all types of triterpene glycosides and phenolic compounds in natural materials can rapidly disintegrate [12]. Meanwhile, high-cost freeze drying is widely regarded as the most effective approach to protect various nutrients because heated-air drying typically results in the destruction of the nutrients despite its cost-effective benefit [13]. Aside from that, Niamnuy et al. 2019 [14] different drying techniques and temperatures affected the drying properties, phenolic and triterpene components, and antioxidant and antibacterial activity of *C. asiatica* extracts are examined. However, no research has been carried out to determine if the deteriorated *C. asiatica* components brought on by the drying process correlate with changes in physiological functions.

Phytochemical investigations on parts of *C. asiatica* indicated the presence of isoprenoids (sesquiterpenes, plant sterols, pentacyclic triterpenoids, and saponins) and phenylpropanoid derivatives (eugenol derivatives, caffeoylquinic acids, and flavonoids) [15]. Of these identified compounds, flavonoids and their derivatives tend to meet a large number of pharmacological requirements. Kaempferol, a highly occurring phenolic compound in the medicinal plants, is also found in this plant in its whole form and as a coumarate derivative. The coumarate derivatives of kaempferol such as castilliferol (3-(4-Hydroxy-trans-cinnamoyloxy)-4’,5,7-trihydroxyflavone) and castillicetin ([2-(3,4-dihydroxyphenyl)-5,7-dihydroxy-4-oxochromen-3-yl]E)-3-(3,4-dihydroxyphenyl)prop-2 enoate) [16] are the least explored compounds in terms of structural activity identification. Structurally, both compounds possess a basic flavonoid skeleton in addition to a cinnamoyloxy unit present in position 4 of the C ring. Both castilliferol and castillicetin differ in the number of hydroxyl units, i.e., for castilliferol four hydroxyl(-OH) units are present in C4’, C5, C7, and C8(cin), whereas for castillicetin six hydroxyl units are present in the C3’, C4’ C5, C7,, C7(cin), and C8(cin) positions.

The goal of the present work is to provide experimental and theoretical evidence on the radical scavenging behavior of selected phytoconstituents present in *C. asiatica* plant extract. In order to accomplish this, the phytoconstituents from harvested *C. asiatica* are extracted using the whole plant. The antioxidant properties of the extract are validated using an experimental procedure. Quantitative analysis of the extract with highest degree of antioxidant activity is also performed via GC-MS and LC-MS. The compounds selected from the extract based on their structural activity are theoretically investigated for their antioxidant capability and compared with the standard kaempferol and its derivatives.

## 2. Materials and Methods

### 2.1. Collection and Drying of C. asiatica

**Figure 1:**
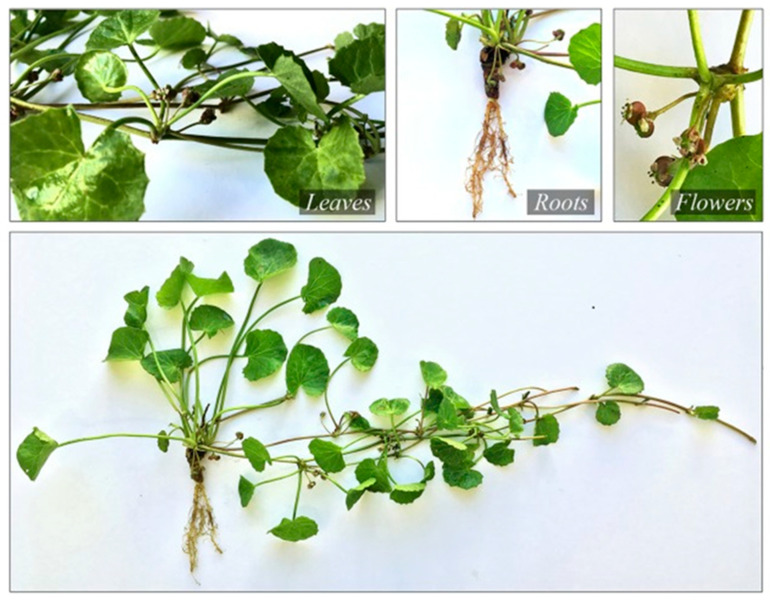
Whole plant of *C. asiatica*.

*C. asiatica* plants were gathered from the tribal settlement of Thootankombai, which is situated in the Western Ghats foothills (11.58185 E, 77.4607 W). A botanical survey of India’s records [17] was used to confirm the plant species. The shoot and leaves (Figure 1) of *C. asiatica* were separated and shade-dried with optimum temperature conditions of about 30 °C. Then, they were shredded into fine powder.

### 2.2. Preparation of Various C. asiatica Extracts

The segregated leaves and shoots of the whole *C. asiatica* were separately extracted under various circumstances after postharvest drying. First, cold water (CW—20 °C) was used to make water extracts. About 20 percent volume (*w*/*v*) of water was added to the dried *C. asiatica*, and the mixture was subjected to extraction by stirring at room temperature for 48 h (CW). EtOH 50% and MeOH were used to prepare the extracts, which were then stirred for 48 h at room temperature (30 °C). To remove insoluble materials, the extracts were individually filtered using filter paper (Whatman, Brentford, UK), centrifuged, and then the remaining excess solvent was evaporated from the final product using a rotary vacuum evaporator and the yield was tabulated (Table 1).

### 2.3. Phytochemical Studies

#### 2.3.1. Determination of TPC and TFC

The total polyphenol content (TPC) present in the prepared extracts was estimated using a slightly altered version of Folin–Ciocalteu’s method. To be precise, 10 μL of extract and 10 μL of sodium carbonate (Na_2_CO_3_ M.W:105.99, 99.5%, purchased from Sigma-Aldrich, St. Louis, MI, USA) at a 2 percent concentration were combined and incubated for 10 min before adding 200 μL of 1 M Folin–Ciocalteu reagent (S.No:1.09001 purchased from Sigma-Aldrich). After 30 min of incubation at room temperature (30 °C), a blue color developed and the absorbance at 750 nm was measured. The total polyphenol content in each extract was calculated as gallic acid equivalent (GAE)/g extract. In a brief procedure, a mixture of 80% ethanol (40 μL), 10% aluminum nitrite (Al(NO_3_)_3_; 99.97% purity, Sigma-Aldrich) (40 μL), potassium acetate (CH_3_CO_2_K; molecular weight: 98.14, 99.0% purity, Sigma-Aldrich) (20 μL), and 80% methanol (150 μL) was combined with 20 μL of the extract. The total flavonoid content was then expressed in mg quercetin equivalent (QE)/g extract by measuring the generated yellow color at 415 nm after it had been allowed to react at room temperature for 10 min.

#### 2.3.2. TLC Analysis

The extract in the mixed extraction solvent was identified via thin-layer chromatography. Identification using TLC was based on glycoside content in *C. asiatica*. The plate used in the process was silica gel GF254 developed with chloroform-MeOH (8:2). The spot was visualized by spraying H_2_SO_4_ (10%) on the plate.

#### 2.3.3. DPPH Assay

A DPPH assay was employed to study the antioxidant property of the prepared plant extracts. Briefly, 0.1 mM concentration of the DPPH was prepared in MeOH. Next, the prepared *C. asiatica* leaves extracts at different concentrations were mixed with the above solution under vigorous stirring at room temperature (30 °C) for 1 h. Ascorbic acid solution prepared in MeOH was set as reference, while the DPPH methanolic solution served as the control. The absorbance value measured using UV-vis spectrophotometer was used to assess the radical scavenging activity. The percentage of inhibition is given by the relation

(1) $$\%\;Inhibition=\frac{Absorbance\left(control\right)-Absorbance(sample)}{Absorbacne(control)}\times 100$$

IC50 is generally defined as the concentration of the extract that decolorizes 50% of DPPH color. A smaller value indicates a better antioxidant property of the extract.

#### 2.3.4. Ferric Reducing Antioxidant Power (FRAP) Assay

A FRAP assay was used to further elucidate the antioxidant efficiency of the prepared extracts. In this procedure, the required concentration of acetate buffer, TPTZ (2,4,6-tris(2-pyridyl)-s-triazine) in diluted hydrochloric acid, and ferric chloride hexahydrate were prepared as stock solutions. The 25 mL of acetate buffer, 2.5 mL of TPTZ, and the ferric chloride hexahydrate solutions were then mixed and retained at 37 °C to prepare the working solution. The *C. asiatica* leaf extracts were dissolved in MeOH and mixed with the Trolox methanolic solution, which was employed as a control. To the test tube holding 2.9 mL of the working solution, 10 μL of MeOH extract solution was added. After that, for 30 min without light, these samples were allowed to interact with one another. The absorbance values were obtained at 593 nm wavelength. Through the use of a standard curve created for varied Trolox concentrations, the FRAP data were translated to micromoles of Trolox equivalents.

#### 2.3.5. ABTS Assay

Potassium persulfate and ABTS solutions were used as the stock solution in the ABTS assay procedure. To generate the working solution, the two stock solutions were combined in exactly equal quantities and maintained at room temperature for 14 h in the dark. By introducing 1 mL of ABTS solution in 60 mL of MeOH, the absorbance value at 734 nm was changed to 0.706 ± 0.01. The appropriate quantity of ABTS solution was added along with the plant extract in various concentrations. After allowing the plant extract and ABTS solution to interact with a reaction time of 1–20 min, the absorbance at 734 nm was measured. As a control, the blank solution that contained solely ABTS solution was used. Ascorbic acid and the extract’s capacity to scavenge ABTS were compared, and the percentage of scavenging activity was inferred by using Equation (1).

#### 2.3.6. GC-MS Analysis

A Perkin-Elmer GC-MS system with a gas chromatograph interfaced to a mass spectrometer (GC-MS) and an Elite-1 fused silica capillary column (30 m × 0.25 mm IDX 1 DF, made entirely of dimethylpolysiloxane) were used to conduct the GC-MS analysis of the methanol extract of *C. asiatica*. The electron ionization device utilized for GC/MS detection had an ionizing energy of 70 eV. With a split ratio of 10:1, a constant flow rate of 1 mL/min, an injection volume of 2 l, and temperatures of 250 °C for the injector and 280 °C for the ion source, helium gas (99.999%) was used as the carrier gas. Further, the chemical constituents identified from the extract were identified by comparing their chromatograms and mass spectral data with those of reference standard compounds from the NIST-09 library.

#### 2.3.7. LC-MS Analysis

Chromatographic and mass spectral analyses of methanol extracts from the plant *C. asiatica* were conducted using an LCMS-8045/8050/8060 (NX) (manufactured by Shimadzu, Kyoto, Japan) and operated in full-scan MSE mode with positive ionization mode. The specific UPLC conditions were as follows: a column with dimensions of 250 mm × 4.6 mm and 5 μm were used. Two eluents were employed: A, which consisted of water with 0.01% formic acid, and B, which contained methanol with 0.01% formic acid. The flow rate was set at 0.2 μL/min, and a 0.2 μL injection volume was used. The calibration mass range for the mass spectrometer was 50–1500 Da. For the mass spectrometric analysis, an electrospray ionization (ESI) source was utilized in positive mode. The source temperature was maintained at 100 °C, while the desolvation temperature was set to 350 °C. Nitrogen was used as the nebulizer gas at a pressure of 45 psi, with a nitrogen desolvation flow rate of 9 L/min. The sampling cone voltage was set to 40 V, the fragmentor voltage to 130 V, and the capillary voltage to 0.5 kV. The scan range for mass detection was *m*/*z* 100–700, with a cycle time of 1 s. Prior to quantification, optimization of both the fragmentor voltage (ranging from 70 to 140 V in 10 V increments) and collision energy (ranging from 25 to 45 eV in 5 eV increments) was performed through parameter ramping in the T-wave collision cell, using ultrahigh-purity argon (≥99.999%) as the collision gas. The optimal settings for collision energy and fragmentor voltage were determined to be 35 eV and 130 V, respectively. Quantitative analysis was carried out in MRM (multiple reaction monitoring) mode, following a method similar to that described by Shen et al., [18] with some modifications.

### 2.4. Computational Detail

**Figure 2:**
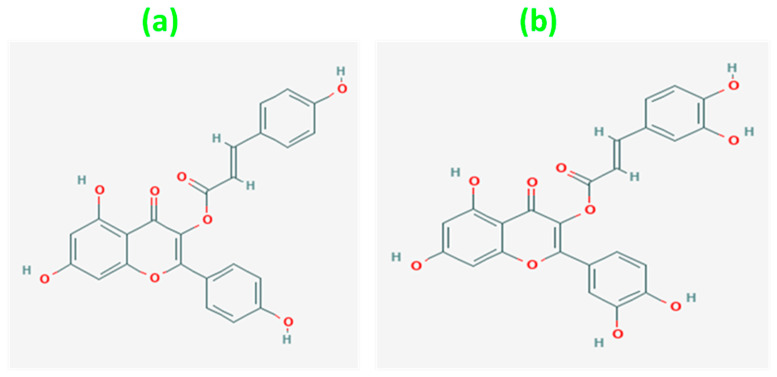
Optimized structures of (**a**) castilliferol and (**b**) castillicetin.

The structural activity of castilliferol and castillicetin was theoretically explored with the aid of density functional theory simulated with the help of Gaussian 16 package [19]. Thermochemical calculations (frequency) were carried out under room temperature with 1 atmospheric pressure indicating an absence of imaginary frequencies. The simulations were carried out using the exchange correlation functional M062X along with triple-zeta basis set 6-311G (d,p) [20]. The molecular structures of the two flavonoids were optimized to attain their corresponding ground = state energy. The ground-state energy of castilliferol was found to be −1526.37757 hartrees, and similarly, for castillicetin, −1676.8523 hartrees was obtained as the ground-state energy [Figure 2]. Bond lengths, bond angles, dihedral angles, and atomic charge values were also obtained from the optimized geometry. The obtained geometry was further used in molecular orbital analysis, electrostatic potential analysis, prediction of molecular properties, Bond Dissociation Enthalpy (BDE) analysis, and target prediction analysis. Lead identification was performed via the SwissADME online database and the same was confirmed using a molecular docking approach supported using the Auto Dock Vina simulation package.

## 3. Results and Discussion

The results of quantitative and qualitative analysis for the *C. asiatica* leaf extract are as follows.

### 3.1. Phytochemical Test Results

Based on the test procedures, polyphenolic tests were conducted. From Table 2, it can be inferred that the EtOH and MeOH fractions favor a high amount of flavonoids and phenolics, which will be reflected in TFC and TPC analysis.

### 3.2. TLC Analysis of C. asiatica Extract

**Figure 3:**
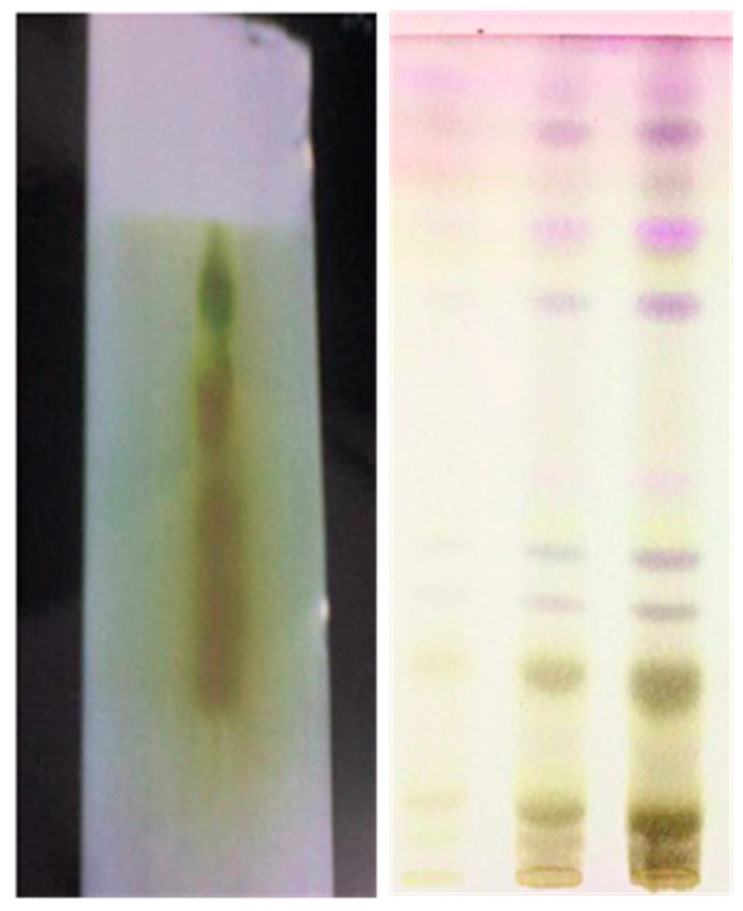
TLC fingerprints of *C. asiatica* extracts.

The glycoside content of *C. asiatica* served as the basis for identification with TLC. Silica gel GF254 was used to create an experiment on the plate. H_2_SO_4_ (10%) was sprayed on the plate to help see the area. The final spot is visible in Figure 3 when illuminated by UV light (366 nm). The dots seen in all samples and standards are visible and observable.

### 3.3. Total Polyphenol and Flavonoid Contents

Table 3 displays the TPC and TFC results. The TFC and TPC levels of the CW extract groups are predicted lower than those of the EtOH and MeOH extract groups. The findings showed that TPC was often dominant in 50% MeOH when compared to other extracts and that in freeze drying, in contrast to heated-air drying, substantially enhanced polyphenols in *C. asiatica* extract. Furthermore, these findings indicated that freeze drying was more effective at enriching TFC than heated-air drying, which was in accordance with a previous report investigating the effect of drying methods on phenolic compounds of daylily flower extracts by [21].

### 3.4. Antioxidant Activity Analysis via Radical Scavenging Assay

In the current investigation, two kinds of free radicals—2-2-diphenyl-1-picrylhydrazyl (DPPH; Sigma-Aldrich, St. Louis, MO, USA) and 2,2′-azino-bis (3-ethylbenzothia-ziline-6-sulfuric acid) (ABTS; Sigma-Aldrich)—were used to evaluate the antioxidant activity of each extract. The ABTS and DPPH radical scavenging activities were assessed using the procedure outlined in [21]. The ability of the extracts to scavenge free radicals was calculated using ascorbic acid equivalent antioxidant capacity (AEAC)/g extract. The ferric reducing ability of plasma (FRAP) activity was used to evaluate each extract’s reducing power using the procedure reported in [21]. The FRAP activity of the extract is given in terms of ascorbic acid equivalent (AAE)/g extract.

From Table 4, it is witnessed that the methanolic extract of *C. asiatica* shows the highest order of scavenging action (IC50 = 65.4, 40.7, 55.7) next to the aqueous extract when compared to its counterpart EtOH.

### 3.5. GC-MS Analysis

**Figure 4:**
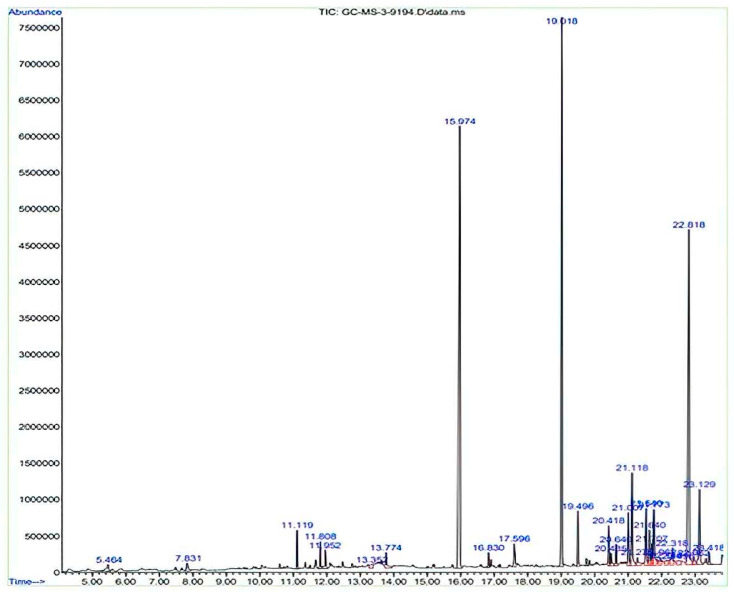
GCMS spectrograph of MeOH extract of *C. asiatica*.

**Figure 5:**
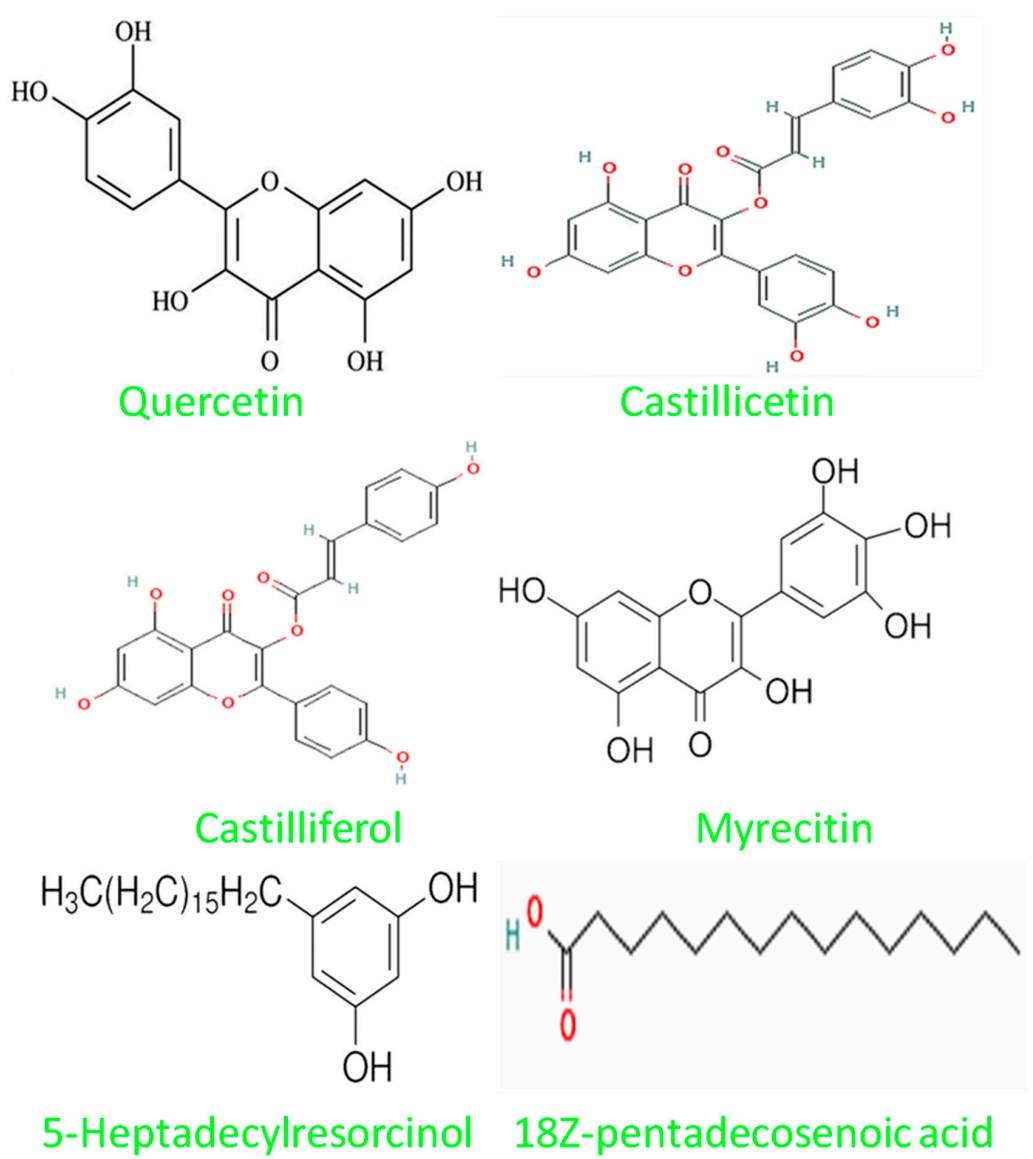
Structures of a few compounds isolated from *C. asiatica* extract.

In the GC-MS analysis of the MeOH extract of *C. asiatica*, 32 chemical constituents (Figure 4) were identified by comparing their chromatograms and mass spectral data with those of reference standard compounds from the NIST-09 library. The classes of compounds identified in this plant (Figure 5) included triterpene glycosides (1), xanthones (1), alkylresorcinols (1), anthocyanins (3), aromatic amide (1), alkaloids (2), polyaromatics (1), flavonoid glycosides (4), phenolic acids (2), coumarin (1), lignans (1), phytosterols (1), prenol lipids (1), saturated fatty acid (1), stilbenes (1), and 10 other compounds that were not identified. Some of the most abundant compounds in this work were quercetin (RT 23.35), castilliferol (RT 23.12), castillicetin (RT 22.8), and myricetin (RT 7.83); an unidentified compound (RT 6.44), 5-Heptadecylresorcinol (RT 5.46), and 18Z-pentadecosenoic acid (RT 13.77) were also present in the GC-MS spectra [22].

### 3.6. LC-MS Analysis

**Figure 6:**
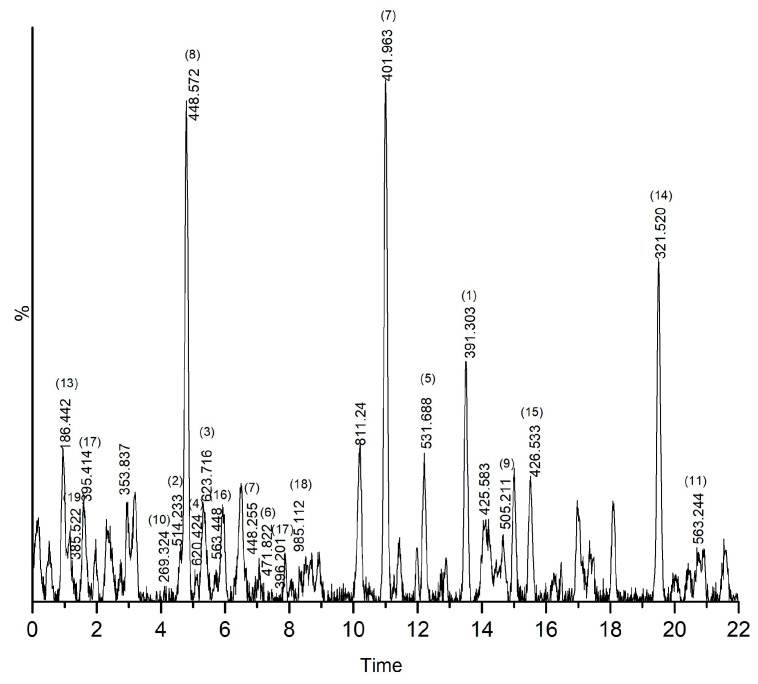
LC-MS chromatogram of methanolic extract of *C. asiatica*.

In the LC-MS analysis of *C. asiatica*, a total of 22 compounds were detected and identified (as shown in Table 5 and Figure 6). The identification of these compounds was based on accurate measurements of their mass and the fragmentation patterns observed [23], which allowed for their structural characterization. These compounds (Table 5) belonged to various chemical classes, including alkaloids (two compounds), anthocyanins (three compounds), flavonoid glycosides (four compounds), coumarin (one compound), polyaromatics (one compound), lignans (one compound), phenolic acids (two compounds), phytosterols (one compound), stilbenes (one compound), triterpene glycosides (one compound), and xanthones (one compound). Additionally, there were five other compounds that were detected but could not be specifically identified. It is worth noting that some of these compound classes have been previously reported in other research studies. Overall, this analysis provided a comprehensive overview of the chemical constituents present in *C. asiatica*, shedding light on its complex composition and potential bioactive compounds.

### 3.7. Theoretical Analysis

#### 3.7.1. Molecular Electrostatic Potential (MEP) Analysis

MEP analysis is an effective approach for analyzing biomolecules’ capacity to give or acquire electrons by viewing the electronegative and electropositive zones surrounding the molecular surface [24].

**Figure 7:**
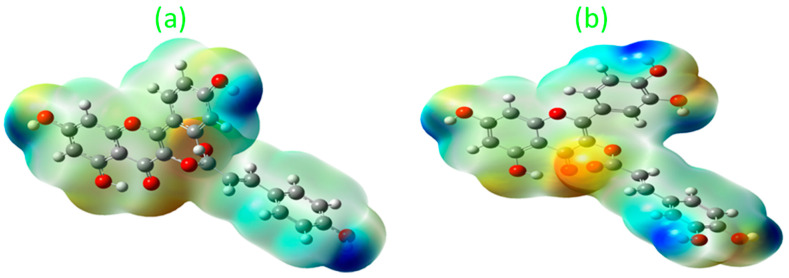
MEP visualization of (**a**) castilliferol and (**b**) castillicetin.

Here, (Figure 7) the flavonoid compounds castilliferol and castillicetin were found to exhibit similar structural features except for hydroxyl units (5′) in the C ring and cinnamoyloxy arm. It was found that, in the two compounds, the hydroxyl unit seems to exhibit the largest electropositive region, thereby determining the compounds’ ability to donate electrons. Meanwhile, the -OH unit in the C5 position is strongly influenced by the electronegative oxygen atom present in the C4 position, making electron donation harder there. When looking over the A, B, and C rings of the flavonoid skeleton, the B ring and cinnamoyloxy ring seem to be highest contributors of electron donation. Based on the electropositive intensity and number of hydroxyl units, castillicetin seems to be more active than castilliferol.

#### 3.7.2. Frontier Molecular Orbital Analysis

**Figure 8:**
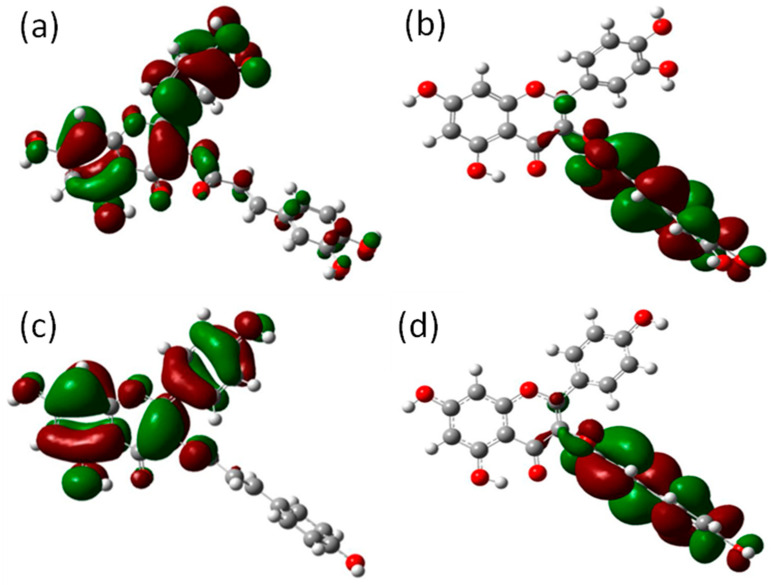
(**a**) Castilliferol HOMO (**b**)Castilliferol LUMO (**c**) Castillicetin HOMO and (**d**) Castillicetin LUMO.

Frontier molecular orbitals are important in a variety of chemical processes because they explain the charge distribution of flavonoids. Electron delocalization in a compound characterizes a molecule’s capacity to donate electrons. In a conceptual sense, the highest occupied molecular orbital (HOMO) is the highest energy orbital that is still occupied, and since it is occupied, it is simpler to remove electrons from this orbital [25]. For castilliferol and castillicetin (Figure 8), the highest occupied orbitals seem to be concentrated in the B ring and A ring, suggesting that these two rings are the active participants in radical scavenging activity, whereas unoccupied orbitals are found to be overlapped over the A ring, since the carbon atoms in the ring are found to attach with oxygen atoms, which exhibit the highest electronegativity. The energy gaps between the occupied energy levels and unoccupied energy levels of castilliferol and castillicetin were found to be 4.06 eV and 3.95 eV.

#### 3.7.3. Global Reactivity Parameters

Molecular descriptors for castilliferol and castillicetin are displayed in Table 6. From the table, it is evident that the ability for electron removal in castillicetin is slightly higher when compared with castilliferol, with an energy difference of 0.066 eV. The electron affinity of both compounds seems to be similar, denoting that there will be no energy change during the removal of atoms from the compounds, and here the difference is −0.043 eV. Here, the hardness of castilliferol is slightly higher when compared to that of castillicetin, with a difference of 0.055 eV and a softness of difference of −0.007 eV, indicating a higher degree of flexibility in terms of reaction for castillicetin. Both compounds have electronegativity, with a difference of 0.011 eV, demonstrating the idea that they easily attract invading radicals and scavenge them. The electrophilicity index of two compounds does not show any major change, reflecting that the two compounds act as donors rather than acceptors, which is an essential quality of the antioxidant compounds. Based on the above results, the minor variations in energy difference do not suggest that castillicetin is better than castilliferol in terms of structural activity, making both of the compounds valid antioxidant compounds.

#### 3.7.4. BDE through H-atom Abstraction

In the HAT mechanism, the hydrogen atom is transferred from a phenolic compound to the free radical RO• to scavenge it, and the mechanism involved is as follows:

K-OH+ RO•→K-O• + ROH (2)

∆HBDE for the HAT mechanism can be calculated using the following equation:

∆H_BDE = H(KO•) + H(ROH)-H(K-OH)-H(RO•) (3)

where the H(KO•), H(ROH), H(K-OH), and H(RO•) are the enthalpies of the phenolic radical, the molecule obtained after hydrogen atom abstraction from the phenolic compound, the starting phenolic compound, and the reactive radical species, respectively. Lower ∆H_BDE values can be attributed to a greater ability of a phenolic compound to donate a hydrogen atom to RO• species [26]. Here, the BDEs are calculated for the gas phase and presented in Table 7.

From the table, it is observed that the BDE values of 4′-OH for castilliferol possess a lower BDE value when compared to the 4′-OH group of castillicetin with an energy difference of 3 k cal/mol. This difference order is 16 k cal/mol for the 5OH site, 9 k cal/mol for 7-OH, and 6 k cal/mol for the 8-OH(cin) site, respectively, for the analyzed compounds. The 3′-OH site and 7-OH site of castillicetin possess BDE difference values of approximately 6 k cal/mol. The reason behind the lowest BDE order of the 4’-OH site of both the compounds is that they are free from any interactions and charge distribution influences posed by the B ring. The 5-OH site attached to the A ring has a BDE of the highest order due to interactions of the hydroxyl unit with electronegative oxygen atoms present nearer to it. Here, the cinnamoxyl unit attached to the 3-OH position of the C ring cannot be considered to be the chief director of structural activity due to the presence of hydroxyl units, which will hold charge distribution towards them to contribute to H-atom abstraction. This observation is in line with the observations made from FMO and MEP analysis.

#### 3.7.5. ADME Analysis

To be successful as a drug, a molecule must be present at the target location in the body for a sufficient amount of time in a bioactive form for the required biological processes to occur. Evaluation of absorption, distribution, metabolism, and excretion (ADME) is an important step in the drug development process. The present work involves physiochemical property and pharmacokinetic property comparisons between two molecules to find an effective drug lead.

The Topological Polar Surface Area (TPSA) (Table 8) value for castillicetin seems to be higher (177.89Å2) than for castilliferol (137.43Å2), making it hard to permeate into cells. Since lipophilicity is a successful and informative physicochemical property in medicinal chemistry, it is a crucial factor in drug discovery and design. To evaluate the lipophilicity characteristic of a compound, Swiss ADME provides five freely available models, i.e., iLogP, XLogP3, WLogP, and MLogP. Log P explains the partition equilibrium of a unionized solute amidst water and an immiscible organic solvent. The larger the log *p* value, the greater the lipophilicity will be. From (Table 8), it is evident that castilliferol seems to possess a higher order of lipophilicity when compared to castillicetin.

Since cytochromes P450 (CYP) play a crucial role in the metabolic biotransformation process that leads to drug clearance, their interaction with the compounds under study is crucial. It is clear that five main isoforms, including CYP1A2, CYP2C19, CYP2C9, CYP2D6, and CYP3A4, are substrates for 50 to 90% of medicinal compounds. It has been proposed that CYP can metabolize small compounds in a synergistic manner to enhance tissue and organism protection. The inhibition of these isoenzymes is undoubtedly a significant factor in drug–drug interactions linked to pharmacokinetics, which can result in toxic or other undesirable side effects due to the buildup and reduced clearance of the drug or its metabolites. Therefore, it is crucial for drug development to forecast a molecule’s potential to inhibit CYPs and result in significant drug interactions as well as to identify which isoforms are affected.

#### 3.7.6. Molecular Target Prediction Analysis

**Figure 9:**
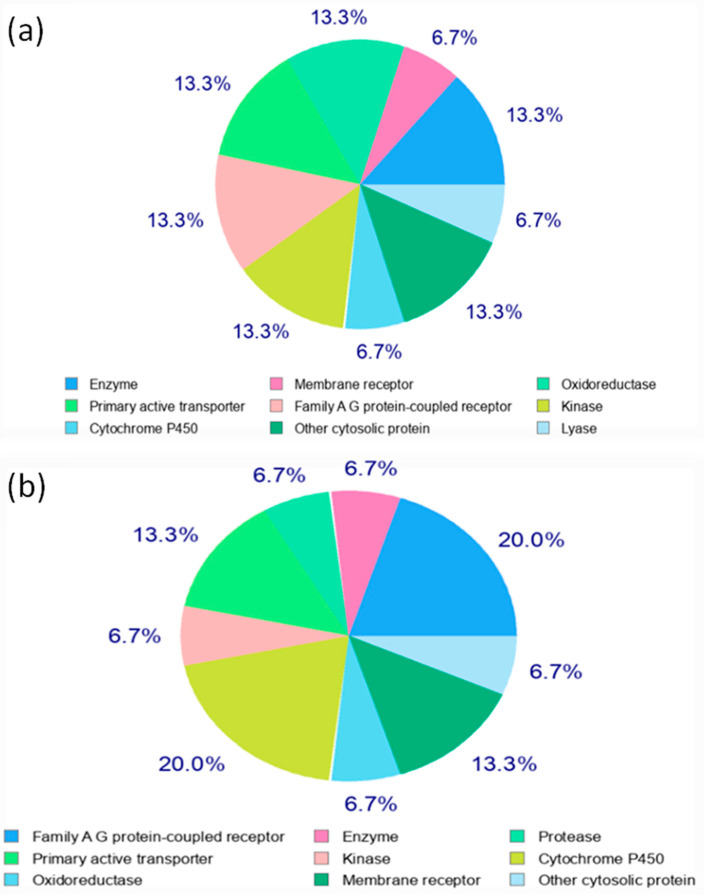
Molecular target prediction report for (**a**) castilliferol and (**b**) castillicetin.

The screening of appropriate targets is a key phase in the drug discovery process. Because the investigated chemicals castilliferol and castillicetin are effective antioxidants, identifying the targets of their interest is critical here. As a result, target prediction analysis was performed using the SwissAdme online database(Figure 9) [27], which selected multiple targets for both drugs, with the top nine targets chosen for interpretation. It has been discovered that enzymatic and protein-linked receptors, oxydoreductase receptors, kinase receptors, and cytosolic receptors are suited for castilliferol. In addition to protein-linked receptors, cytochrome P450 is a target of interest for castillicetin. Castillicetin appears to have a higher percentage of target screening than castilliferol.

#### 3.7.7. Molecular Docking Analysis

**Figure 10:**
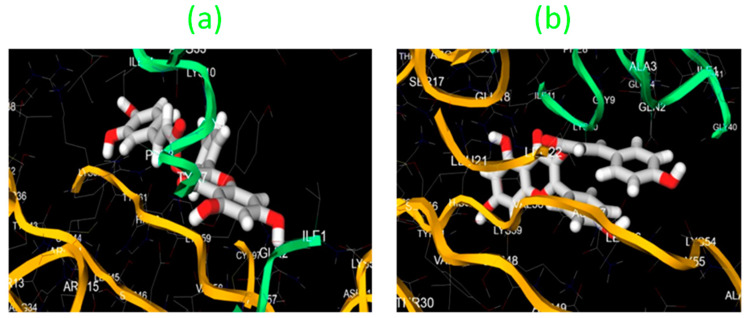
Highest binding affinity docking positions for (**a**) castillicetin and (**b**) castilliferol.

According to the docking results for castilliferol and castillicetin, the biological activity of different molecules can be anticipated based on the energy (kcal/mol), number of contacts, and amino acids involved in the ligand–protein interaction. For in silico docking experiments and to check their compatibility with enzymatic receptor 1IR3, Auto DockVina software (version 1.1.2) was employed. The best compounds were expected to have the highest interactions with the receptor and minimal ligand–receptor binding energy (six bond lengths). The results from docking studies displaying the ideal docking positions are shown in Figure 10. Compounds that interact with enzymatic receptors may be effective at reducing blood sugar levels by modulating insulin signaling. As shown by the lowest interaction energy, bond length, and number of ligand–protein interactions, castillicetin and castilliferol were projected to be the most effective compounds (Figure 9). As a result of their extraordinary 1IR3 interaction, these compounds can be regarded as possible diabetes-fighting agents since they directly affect the insulin receptor. Before drawing any incorrect conclusions, these compounds need to be thoroughly examined in additional in vitro and in vivo tests.

## 4. Conclusions

The TPC and TFC of the aqueous, methanolic, and ethanolic extracts of *C. asiatica* plant have been determined. The spots obtained from the TLC plates also depict the same observation made in the previous analysis. The GC-MS analysis presented the mass percentage of various flavonoids, which include castilliferol and castillicetin. The LC-MS profile also depicts the majority amounts of the compounds confirmed via GC-MS analysis. These compounds are further considered for theoretical analysis to elucidate their SAR. Castilliferol and castillicetin were found to exhibit similar structural features except for hydroxyl units (5′) in the C ring and cinnamoyloxy arm. It was found that for the two compounds, the hydroxyl unit seems to exhibit the largest electropositive region, thereby denoting their ability to donate electrons. Meanwhile, the –OH unit in the C-5 position is strongly influenced by the electronegative oxygen atom present in the C-4, position making electron donation harder there. When looking over the A, B, and C rings of the flavonoid skeleton, the B ring and cinnamoyloxy ring seem to be the highest contributors of electron donation. Based on the electropositive intensity and number of hydroxyl units, castillicetin seems to be more active than castilliferol. ADME analysis suggests that castilliferol is an effective drug lead since it has fewer violations than castillicetin. From the molecular target prediction analysis, it was found that both the molecules prefer enzymatic targets. Hence, a molecular docking analysis was performed, which shows that the binding affinity of castilliferol with the target is superior when compared with castillicetin. Based on the above results, it is suggested that among the two flavonoids of *C. asiatica*, castilliferol is more structurally active than its counterpart castillicetin.

## Figures and Tables

**Table 1:** Yield during extraction with solvents of increasing polarity.

Solvent	*C. asiatica* Extract
CW	60.1 ± 1.12
MeOH	19.5 ± 1.0
EtOH	28.4 ± 0.11

**Table 2:** Test for the presence of phytochemicals.

Extract	Test			
	Flavonoids	Phenolics	Tannins	Alkaloids
H_2_O	+	+	++	++
MeOH	+++	+++	++	++
EtOH	+++	+++	+	+

**Table 3:** Determination of TPC and TFC.

Extract	Polyphenol (GAE mg/g)	Flavonoid (QE mg/g)
CW	34.4 ± 1.1	7.5 ± 0.1
MeOH	132.6 ± 8.4	15.1 ± 1.4
EtOH	106.2 ± 7.5	14.8 ± 0.3

Values are the mean of three independent analyses of the extract ± SD (n = 3).

**Table 4:** Free radical scavenging data for *C. asiatica* extract.

Extract (AEAC mg/g)	Radical Scavenging Activity (IC_50_)		FRAP (AE mg/g)
	ABTS	DPPH	
CW	46.5 ± 0.8	21.9 ± 1.1	34.1 ± 0.6
MeOH	65.4 ± 1.2	40.7 ± 1.2	55.7 ± 0.9
EtOH	66.6 ± 0.3	37.2 ± 1.2	56.3 ± 1.2

Values are mean of three independent analyses of the extract ± SD (n = 3).

**Table 5:** LC-MS profile of methanolic extract of *C. asiatica*.

S. No	Compound	TIC ^t^R(min)	M + ^H^(*M*/*Z*)	CID Product ^ions^ (*M*/*Z*)
	Alkaloid			
1	Dioncopeltine A	1.15	395	274,122
2	Dipyridamole	4.258	514	265,186,142
	Anthocyanins			
3	Delphinidin 3,5-O-diglucoside	5.223	623	409,307
4	Delphinidin 3-O-glucosyl-glucoside	5.242	620	413,307
5	Pelargonidin-3-O-(6”-malonyl-glucoside)	12.412	531	375,353,243
	Flavonoid Glycosides			
6	Isorhamnetin 3-O-rutinoside	7.122	471	287,145
7	Kaempferol 7-O-glucoside	5.162	448	402,307
8	Dihydroquercetin 3-O-rhamnoside	7.427	448	333,145
9	Isorhamnetin 3-O-glucuronide	14.64	505	441,163
	Coumarin			
10	Coumestrol	4.615	269	163
	Polyaromatic			
11	6,13-Dihexyl-2,3,9,10-termethylpentacene- 1,4,8,11-tetrone	4.921	563	411,307,209
	Lignans			
12	Todolactol A	4.422	377	163,145
	Phenolic Acid			
13	Dihydrocaffeic acid	1.002	186	139
14	Cinnamoyl glucose	4.025	311	292,166
	Phytosterols			
15	Stigmasterol	14.18	425	413,
	Lipids			
16	Delta-carotene-1,2-epoxide	21.68	563	453,317,203
	Stilbenes			
17	Resveratol 3-O-glucoside	7.28	396	309,189,171
	Triterpene Glycoside			
18	Ziziphin	8.115	985	453,291,154
	Xanthones			
19	8-Desoxygartatin	1.54	385	248,203,182

**Table 6:** Molecular descriptive parameters for castilliferol and castillicetin.

Molecular Descriptors	Eₒ (eV) of Castilliferol	Eₒ(eV) of Castillicetin
IP (eV)	6.013	5.947
EA(eV)	1.953	1.996
ω(eV)	2.030	1.975
S(eV)	0.246	0.253
X(eV)	3.983	3.972
ɳ(eV)	3.908	3.993

**Table 7:** Gas phase BDEs of castilliferol and castillicetin.

-OH Sites	Castilliferol (k cal/mol)	Castillicetin (k cal/mol)
3’OH	-	41.85
4’OH	37.23	40.34
5OH	58.54	74.21
7OH	44.28	53.26
7OH(cin)	-	46.24
8OH(cin)	41.24	47.38

**Table 8:** (**a**) Physiochemical properties and lipophilicity of castilliferol and castillicetin. (**b**) Pharmacokinetic properties of castilliferol and castillicetin.

**(a)**					
**Molecule**	**TPSA**	**iLogP**	**XLogP3**	**WLogP**	**MLogP**
Castilliferol	137.43	2.67	4.36	3.79	1.17
Castillicetin	177.89	1.83	3.65	3.2	0.16
**(b)**					
**Molecule**	**CYP1A2 Inhibitor**	**CYP2C19 Inhibitor**	**CYP2C9 Inhibitor**	**CYP2D6 Inhibitor**	**CYP3A4 Inhibitor**
Castilliferol	No	No	Yes	No	No
Castillicetin	Yes	No	Yes	No	No

## Data Availability

Not applicable.
