# Correction: Kandasamy et al. Phytochemical Analysis and Antioxidant Activity of Centella Asiatica Extracts: An Experimental and Theoretical Investigation of Flavonoids. *Plants* 2023, *12*, 3547

**DOI:** 10.3390/plants15101466

**Published:** 2026-05-12

**Authors:** Anbazhakan Kandasamy, Kanakaraj Aruchamy, Praveena Rangasamy, Deepha Varadhaiyan, Chandrasekar Gowri, Tae Hwan Oh, Subramaniyan Ramasundaram, Balasankar Athinarayanan

**Affiliations:** 1 Department of Physics, Gobi Arts & Science College, Gobichettipalayam, Erode 638453, India; 2 School of Chemical Engineering, Yeungnam University, Gyeongsan 38541, Republic of Koreataehwanoh@ynu.ac.kr (T.H.O.); 3 Department of Chemistry, Bannari Amman Institute of Technology, Sathyamangalam 638401, India; deephav@bitsathy.ac.in; 4 Sri Shanmugha College of Engineering and Technology, Pullipalayam, Salem 637304, India

In the original publication [1], there was a mistake in Figure 6 as published. One of the authors, Anbazhakan Kandasamy, omitted a proper citation for the LC-MS chromatogram reported by Ondeki et al. To rectify this, the original LC-MS spectrum (represented in Table 5) has been replaced in Figure 6 by his experimentally obtained LC-MS spectrum. The corrected Figure 6 appears below. The authors state that the scientific conclusions are unaffected. This correction was approved by the Academic Editor. The original publication has also been updated.

## Figures and Tables

**Figure 6:**
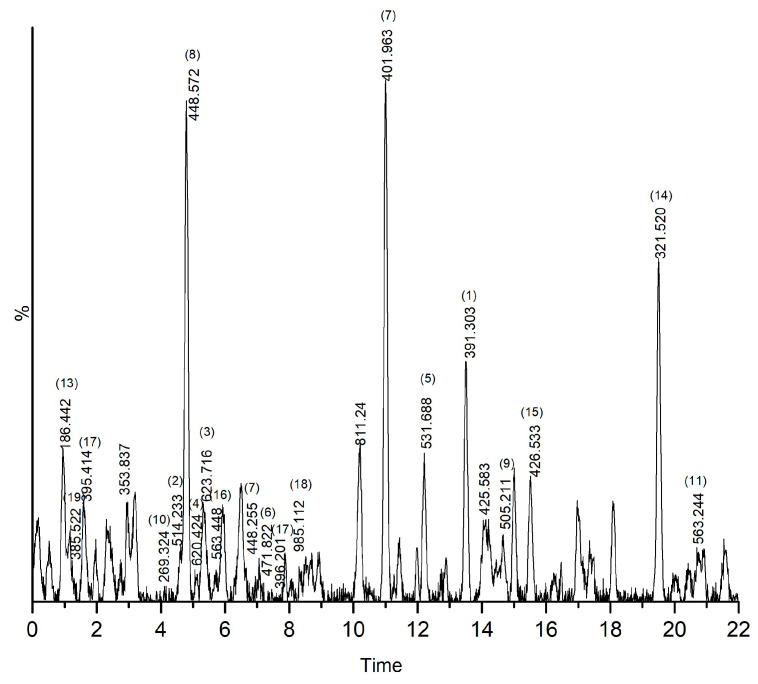
LC-MS chromatogram of methanolic extract of *C. asiatica*.
